# Exploring Factors Influencing Stroke Risk: Insights From a Predictive Analysis

**DOI:** 10.7759/cureus.67976

**Published:** 2024-08-27

**Authors:** Venkatram Murugesan, Murugesan Natesan, Vahidha Sulthana, Pranav R Donapaty

**Affiliations:** 1 Nephrology, East Coast Hospitals, Pondicherry, IND; 2 Biology, Eastlake High School, Sammamish, USA

**Keywords:** fasting, stroke, cholesterol, glucose, insulin, age

## Abstract

Introduction

Stroke is a serious medical condition characterized by the sudden interruption of blood flow to the brain, resulting in the death of brain cells. It is a leading cause of long-term disability and mortality worldwide. Stroke has some associated risk factors, both modifiable and non-modifiable ones. As for non-modifiable risk factors, these are age, gender (men are more vulnerable), and family history of stroke. The controllable or adjustable risk factors include hypertension (high blood pressure), diabetes, smoking, high cholesterol levels, obesity, and insulin resistance.

Methods

In our study, we collected data from 229 patients which were originally collected for clinical purposes and were retrospectively analyzed. These data contain features such as sex and age, the presence of ischemic heart disease (IHD) or stroke history, and different blood sugar readings. These measurements include fasting blood sugar (FBS), postprandial blood sugar (PPBS), HbA1c%, and insulin levels (fasting and postprandial). Furthermore, cholesterol was also tested, such as total cholesterol, triglycerides (TGL), high-density lipoprotein (HDL), low-density lipoprotein (LDL), and very low-density lipoprotein (VLDL). Surprisingly, stroke was observed in 24 of the 205 patients. This contrast permits us to be concerned with the chance of the association between stroke and insulin levels. Given the imbalanced nature of our outcome variable (stroke occurrence), the primary analytical method will be logistic regression.

Results

In this cross-sectional study, we investigated the association between high insulin levels (both fasting and postprandial) and the occurrence of stroke within a dataset of 229 patients. Out of the 229 included cases, 102 individuals were female (44.5%) and 127 individuals were male (55.5%). Twenty-four cases have ischemic heart disease (10.5%). Among the analyzed cases, 24 individuals have a history of stroke. The average age of the sample is approximately 57 years ± 14.87. There was no significant difference between the males and females in most of the descriptive statistics. However, females experienced significantly higher levels of postprandial glucose level and significantly lower levels of postprandial insulin. According to our predictive model, we found that an increase in fasting insulin levels was linked to a lower risk of stroke occurrence. On the other hand, increasing insulin postprandial levels and age were associated with an increased risk of stroke.

Conclusion

Our study identified age, fasting insulin, and postprandial insulin as key factors influencing stroke risk. Higher fasting insulin levels were associated with reduced risk, while increased postprandial insulin and age were linked to higher risk. Blood glucose, cholesterol, and triglycerides had minor effects. Notably, higher total cholesterol and triglyceride levels were slightly associated with lower stroke occurrence. Further research with larger samples is needed for validation.

## Introduction

Stroke is a clinically diagnosed syndrome in which focal neurological deficits appear as a result of cerebral circulation dysfunction or vascular cognitive disorder [1]. Stroke is one of the most common causes of disability and death worldwide. It is the second most common cause of mortality in middle to high-income countries. Stroke itself is not a single disease entity but instead is caused by many risk factors and pathological processes [2,3]. Age is an important risk factor for stroke. During the process of aging, the arteries that supply blood become narrower and less flexible. This increases the possibility of obstruction or rupture and eventually stroke. In the same way, diseases such as hypertension, diabetes, and atrial fibrillation are much more common in the elderly, increasing the risk of stroke. The incidence of stroke doubles in each decade after the age of 50 years [4,5].

Ischemic heart disease (IHD) is considered another risk factor for stroke. In both conditions, the underlying mechanisms are similar. Having IHD essentially means there's already a problem with blood vessel health, making the brain even more vulnerable to a stroke event [6]. Atherosclerosis is the most important risk factor for both conditions. It results from the accumulation of fatty streaks in the medium-sized and large arteries. Plaque buildup results in narrowing and hardening of arteries, a condition that hinders the blood supply to the brain, which can lead to ischemic stroke. Furthermore, this plaque deposition weakens the vessel wall leading to ruptures and hemorrhagic strokes [7-9].

Hyperglycemia can increase the process of atherosclerosis, promote inflammation, and lead to oxidative stress. Diabetes is considered an independent risk factor for stroke [10]. The incidence of stroke increases twofold in uncontrolled diabetes. Furthermore, stroke is responsible for 20% of all mortality cases in diabetics [11]. The nature of the association between dyslipidemia and stroke is complex. The risk of ischemic stroke is increased by the increasing amount of total cholesterol, but it is also decreased by having high-density lipoprotein-cholesterol [12,13]. As for total cholesterol, its levels are in a negative correlation with ICH risk. The use of statins in secondary prevention following a stroke appears to reduce the risk of ischemic stroke (as well as functional outcome and mortality) with no definite increase in the risk of intracerebral hemorrhage [11]. Previous studies have indicated that diabetes and insulin resistance are significant risk factors for stroke. Hyperinsulinemia, a condition marked by elevated insulin levels, has been associated with an increased risk of cardiovascular events, including stroke.

Research by Xun et al. demonstrated that higher fasting insulin concentrations are linked to an increased risk of hypertension and ischemic heart disease, but the association with stroke remains less clear [14]. Thacker et al. found that post-glucose load measures of insulin resistance, rather than fasting insulin levels, were associated with an increased risk of ischemic stroke in non-diabetic elderly individuals [15]. Given these mixed findings, our study aimed to examine the risk of stroke by analyzing the association between the presence of stroke and multiple metabolic variables, with a particular focus on the significant role of insulin levels (both fasting and postprandial) in stroke occurrence. By leveraging a comprehensive dataset from East Coast Hospitals, Pondicherry, India, we aim to provide more definitive insights into how insulin levels impact stroke risk.

The aim of this study was to examine the risk of stroke by analyzing the association between the presence of stroke and multiple metabolic variables, with a particular focus on the significant role of insulin levels (both fasting and postprandial) in stroke occurrence. The objectives were to analyze the association between various metabolic risk factors (including fasting blood sugar, postprandial blood sugar, HbA1c, cholesterol levels, and insulin levels) and stroke occurrence, to identify significant predictors of stroke risk among these variables, and to adjust for potential confounding factors to ensure the robustness of the observed associations.

## Materials and methods

This observational study employs a retrospective cohort design to investigate the association between high insulin levels (both fasting and postprandial) and the occurrence of stroke. The study utilizes existing patient data from medical records, aiming to identify potential associations between metabolic factors and stroke occurrence. The analysis focuses on a single time point, leveraging the de-identified dataset from East Coast Hospitals, Pondicherry, India.

Study population

The study population comprises 229 patients treated at East Coast Hospitals. These patients' medical records were retrospectively reviewed to extract relevant data for analysis. The dataset includes patients with a range of metabolic markers and cardiovascular events, specifically focusing on those who have experienced a stroke compared to those who have not.

Patients were included in the study if their medical records contained complete data on the following variables: age, sex, IHD, stroke, fasting blood sugar (FBS), postprandial blood sugar (PPBS), HbA1c%, insulin levels (fasting and postprandial), total cholesterol, triglycerides (TGL), high-density lipoprotein (HDL), low-density lipoprotein (LDL), and very low-density lipoprotein (VLDL). Additionally, patients had to have been diagnosed and treated for various cardiovascular conditions at East Coast Hospitals and have a documented history of stroke in their medical records. Patients were excluded from the study if they had incomplete or missing data on key variables if their records did not clearly indicate the presence or absence of a stroke, or if they had secondary or non-primary stroke causes that could confound the analysis, such as traumatic brain injury.

Sample size estimation

Formula used: n=(Zα/2+Zβδ)2×2σ2n = \left( \frac{{Z_{\alpha/2} + Z_{\beta}}}{{\delta}} \right)^2 \times 2\sigma^2n=(δZα/2​+Zβ​​)2×2σ2

After putting the above values in the formula, the total number of sample size will be 150. In the present study, for sample size estimation, we used an effect size of 12.4, power of 0.9, and an alpha value of 0.01, resulting in a minimum sample size of 150 (adapted from [16]).

Key considerations

Handling Imbalance

The disparity between the number of stroke and non-stroke cases is a notable aspect of our dataset. While this may limit the statistical power to detect small effects, logistic regression remains an appropriate tool for exploring associations in such datasets. Care will be taken to avoid overusing and selecting confounders for inclusion in the model.

Model Specification

The logistic regression model will be specified to include high insulin levels as the primary independent variable of interest, with stroke occurrence as the binary dependent variable. We will include covariates based on their potential to confound the relationship between insulin levels and stroke risk, informed by existing literature and biological plausibility.

Statistical approach

The statistical analysis for this study involved several comprehensive steps to understand the associations between metabolic variables and stroke occurrence. Initially, descriptive statistics were conducted to summarize the distribution of all variables, including age, sex, IHD, stroke history, FBS, PPBS, HbA1c%, insulin levels (fasting and postprandial), total cholesterol, TGL, HDL, LDL, and VLDL. Means and standard deviations were calculated for continuous variables, while frequencies and percentages were computed for categorical variables. Following this, bivariate analyses were performed to explore the associations between stroke occurrence and each independent variable. Independent t-tests were used to compare means between stroke and non-stroke groups for continuous variables, while chi-square tests assessed associations for categorical predictors. Univariate logistic regression analyses were conducted for each predictor variable to examine their individual relationships with stroke occurrence, with odds ratios (ORs) and 95% confidence intervals (CIs) calculated. Subsequently, a multivariate logistic regression model was constructed to assess the independent effects of multiple predictor variables on stroke risk. This model included high insulin levels (fasting and postprandial) as the primary independent variables, with stroke occurrence as the binary dependent variable. Covariates such as age, sex, and other metabolic markers (FBS, PPBS, HbA1c, total cholesterol, TGL, HDL, LDL, VLDL, and IHD) were included based on their potential to confound the relationship between insulin levels and stroke risk, informed by existing literature and biological plausibility. Adjusted odds ratios (AORs) and 95% CIs were calculated to account for these confounders. Given the imbalanced nature of our outcome variable (24 stroke cases out of 229 patients), particular attention was given to ensuring the robustness of the logistic regression model. Methods such as weighting or using robust standard errors were considered to address potential issues arising from the imbalance in the dataset. The logistic regression model's goodness-of-fit was assessed using appropriate statistical tests, and model diagnostics were performed to check for multicollinearity and other assumptions.

## Results

In this cross-sectional study, we investigated the association between high insulin levels (both fasting and postprandial) and the occurrence of stroke within a dataset of 229 patients. Out of the 229 included cases, 102 individuals were female (44.5%) and 127 individuals were male (55.5%). Twenty-four cases have IHD (10.5%). Among the analyzed cases, 24 individuals have a history of stroke. The average age of the sample is approximately 57 years ± 14.87. The mean FBS was 136.3 mg/dL± 58.3. Similarly, the PPBS averages at 242 mg/dL ± 111.3. The mean value of HbA1C was 7.5 ± 2.1. The fasting insulin averages around 20.5 µU/mL, with a standard deviation of about 21.36 µU/mL, while post-prandial insulin averages approximately 126 µU/mL, with a standard deviation of 151.05 µU/mL. Total cholesterol averages 151.7 mg/dL, 51.7 mg/dL, while HDL cholesterol averages 36.59 mg/dL, with a standard deviation of approximately 13.8 mg/dL. Qualitative data were described as n (%), while the quantitative data were represented as mean and standard deviation. The remaining data of descriptive statistics is reported in Table 1.

**Table 1:** Descriptive statistics of the included patients

Variables	Frequency	Percent
Gender (Females / Males)	102 / 127	44.5 / 55.5
Ischemic Heart Disease	24	10.5
Stroke	24	10.5
Variables	Mean	SD
Age (years)	57.24	14.867
Fasting Blood Sugar (FBS)	136.371	58.3032
Postprandial Blood Sugar (PPBS)	242.048	111.311
Hemoglobin A1c (HbA1c%)	7.577	2.1806
Insulin - Fasting	20.51509	21.35771
Insulin - Postprandial	126.0401	151.0544
Total Cholesterol	151.729	51.7083
Triglycerides (TGL)	135.563	72.361
High-Density Lipoprotein (HDL)	36.59	13.803
Low-Density Lipoprotein (LDL)	88.06	43.41
Very Low-Density Lipoprotein (VLDL)	26.41	13.102

Stratification of the descriptive data according to gender

The average female age is 57.03 years ± 14.45 years. The mean age of males is 57.42 years±15.25 years with no significant difference between both groups with p-value =0.8. The average FBG level of the female is 148.06 and that of the male is 126.98 with p-value =0.06. The average PPBS level is 258.77 ±121.12 mg/dl for the female group and 228.62±101.26 mg/dl for the male group. In this dataset, we have a p-value that is 0.04, and it is a statistically significant difference in PPBS between men and women. HbA1c: Hemoglobin A1c gives an indication of blood sugars’ long-term control. The average HbA1c for females is 7.85 which is different from 7.36 for males. A higher standard deviation is observed for females at 2.24 than for males at 2.12. There is a p-value of 0.09 which suggests that there is no statistically significant difference in HbA1c between men and women in the dataset presented.

Fasting Insulin

The average fasting insulin level for females is 20.1 µIU/mL with a standard deviation of 20.9 µIU/mL. The average male fasting insulin level is 20.84 µIU/mL with a standard deviation of 21.77 µIU/mL. The p-value is 0.8 which means there is no statistically significant difference in fasting insulin levels between the sexes in this dataset. 

Postprandial Insulin

The average postprandial insulin level for females is 98.8µIU/mL with a standard deviation of 102.2 µIU/mL. The average male postprandial insulin level is 147.88 µIU/mL with a standard deviation of 178.42 µIU/mL with a p-value of 0.01indicating a significant difference between both groups (Table 2).

**Table 2:** Stratification of the descriptive data according to gender Data represented as mean and SD. * indicates a significant difference according to the independent samples test. p-value <0.01 is significant.

Variable	Sex	p-value
Age (years)	Female	0.8
	Male	
Fasting Blood Sugar (FBS)	Female	0.06
	Male	
Postprandial Blood Sugar (PPBS)	Female	0.04*
	Male	
Hemoglobin A1c (HbA1c%)	Female	0.09
	Male	
Insulin - Fasting	Female	0.8
	Male	
Insulin - Postprandial	Female	0.01*
	Male	
Total Cholesterol	Female	0.7
	Male	
Triglycerides (TGL)	Female	0.3
	Male	
High-Density Lipoprotein (HDL)	Female	0.07
	Male	
Low-Density Lipoprotein (LDL)	Female	0.9
	Male	
Very Low-Density Lipoprotein (VLDL)	Female	0.7
	Male	

Stratification of the descriptive data according to having a stroke

Age

The stroke group has a mean age of 64.38 years which is significantly higher than the control group (56.41 years) where the p-value is 0.002.

Blood Sugar

FBS: The two groups tested have not shown any statistically significant difference (p-value = 0.18). Regarding the FBS, the difference in the PPBS between the stroke group 267.75±122.4 and the control group 239.03±109.8 does not reach the level of statistical significance either (p-value = 0.23).

HbA1c

In this way, the stroke group reaches an average (8.075%) but the difference amount is not statistically significant (p-value = 0.23).

Insulin Levels

Fasting and insulin levels after food intake show no statistically significant difference across groups (p-value was 0.93 and 0.51, respectively).

Lipids

Total cholesterol levels between the groups show minimal disparity, with Group 1 exhibiting a mean of 144.9 mg/dL and Group 2 at 152.5 mg/dL, a difference deemed statistically insignificant (p = 0.49). Similarly, triglyceride levels display no substantial variance, as Group 1 averages 117.02 mg/dL compared to Group 2's 137.7 mg/dL, with a non-significant p-value of 0.18.HDL levels present minor differences, with Group 1 at 40.38 mg/dL and Group 2 at 36.15 mg/dL, yet again, statistically insignificant (p = 0.15). Likewise, the comparison of LDL levels reveals no significant distinction, with Group 1 at 81.04 mg/dL and Group 2 at 88.8 mg/dL, yielding a p-value of 0.4 (Table 3).

**Table 3:** Stratification of the descriptive data according to the history of stroke p-value <0.01 is significant. Data represented as mean and SD. * indicates a significant difference according to the independent samples test.

Variable	Stroke Group	Control Group	p-value
Age	64.38 ± 10.5	56.41 ± 15	0.002*
Fasting Blood Sugar (FBS)	151.41 ± 62.5	134.61 ± 57.6	0.18
Postprandial Blood Sugar (PPBS)	267.75 ± 122.4	239.03 ± 109.8	0.23
Hemoglobin A1c (HbA1c%)	8.075 ± 2.4	7.5 ± 2.1	0.23
Insulin - Fasting	20.18 ± 13.6	20.5 ± 22.1	0.93
Insulin - Postprandial	106.92 ± 128.3	128.2 ± 153.6	0.51
Total Cholesterol	144.9 ± 36.7	152.5 ± 53.2	0.49
Triglycerides (TGL)	117.02 ± 43.5	137.7 ± 74.7	0.18
High-Density Lipoprotein (HDL)	40.38 ± 13.7	36.15 ± 13.7	0.15
Low-Density Lipoprotein (LDL)	81.04 ± 33.3	88.8 ± 44.4	0.4
Very Low-Density Lipoprotein (VLDL)	23.38 ± 8.7	26.76 ± 13.4	0.23
Variable	Stroke Group	Control Group	P-value
Age	64.38 ± 10.5	56.41 ± 15	0.002*
Fasting Blood Sugar (FBS)	151.41 ± 62.5	134.61 ± 57.6	0.18
Postprandial Blood Sugar (PPBS)	267.75 ± 122.4	239.03 ± 109.8	0.23
Hemoglobin A1c (HbA1c%)	8.075 ± 2.4	7.5 ± 2.1	0.23
Insulin - Fasting	20.18 ± 13.6	20.5 ± 22.1	0.93
Insulin - Postprandial	106.92 ± 128.3	128.2 ± 153.6	0.51
Total Cholesterol	144.9 ± 36.7	152.5 ± 53.2	0.49
Triglycerides (TGL)	117.02 ± 43.5	137.7 ± 74.7	0.18
High-Density Lipoprotein (HDL)	40.38 ± 13.7	36.15 ± 13.7	0.15
Low-Density Lipoprotein (LDL)	81.04 ± 33.3	88.8 ± 44.4	0.4
Very Low-Density Lipoprotein (VLDL)	23.38 ± 8.7	26.76 ± 13.4	0.23

Predictive model of variables that may be associated with stroke

A logistic regression was performed to ascertain the effect of insulin (fasting and postprandial), age, FBS, PPBS, HbA1c total cholesterol, TGL, HDL, LDL, VLDL, sex and IHD on the likelihood that participants had a stroke. The logistic regression model was statistically significant. The variables most associated with stroke seem to be age, insulin fasting, and postprandial. An increasing value of fasting insulin was associated with a reduction in the likelihood of exhibiting stroke. On the other hand, an increasing value of postprandial insulin and age was associated with an increased likelihood of exhibiting stroke (Table 4). 

**Table 4:** Logistic regression between different independent variables and stroke outcome CI: Confidence interval

Variable	B	Exp(B)	95% CI for EXP(B)
Age in years	0.038	1.039	1.003 - 1.077
Fasting Blood Sugar (FBS)	0	1.000	0.988 - 1.012
Post-Prandial Blood Sugar (PPBS)	0.002	1.002	0.996 - 1.008
Hemoglobin A1c (HbA1c)	0.07	1.072	0.825 - 1.394
Insulin - Fasting	-0.111	0.895	0.806 - 0.993
Insulin - Postprandial	0.024	1.024	1.002 - 1.047
Total Cholesterol	-0.017	0.984	0.913 - 1.060
Triglycerides (TGL)	-0.017	0.983	0.937 - 1.031
High-Density Lipoprotein (HDL)	0.03	1.030	0.953 - 1.114
Low-Density Lipoprotein (LDL)	0.014	1.014	0.940 - 1.093
Very Low-Density Lipoprotein (VLDL)	0.078	1.081	0.840 - 1.393
Ischemic Heart Disease (IHD)	0.677	1.969	0.371 - 10.438
Sex	0.376	1.457	0.568 - 3.735

**Figure 1:**
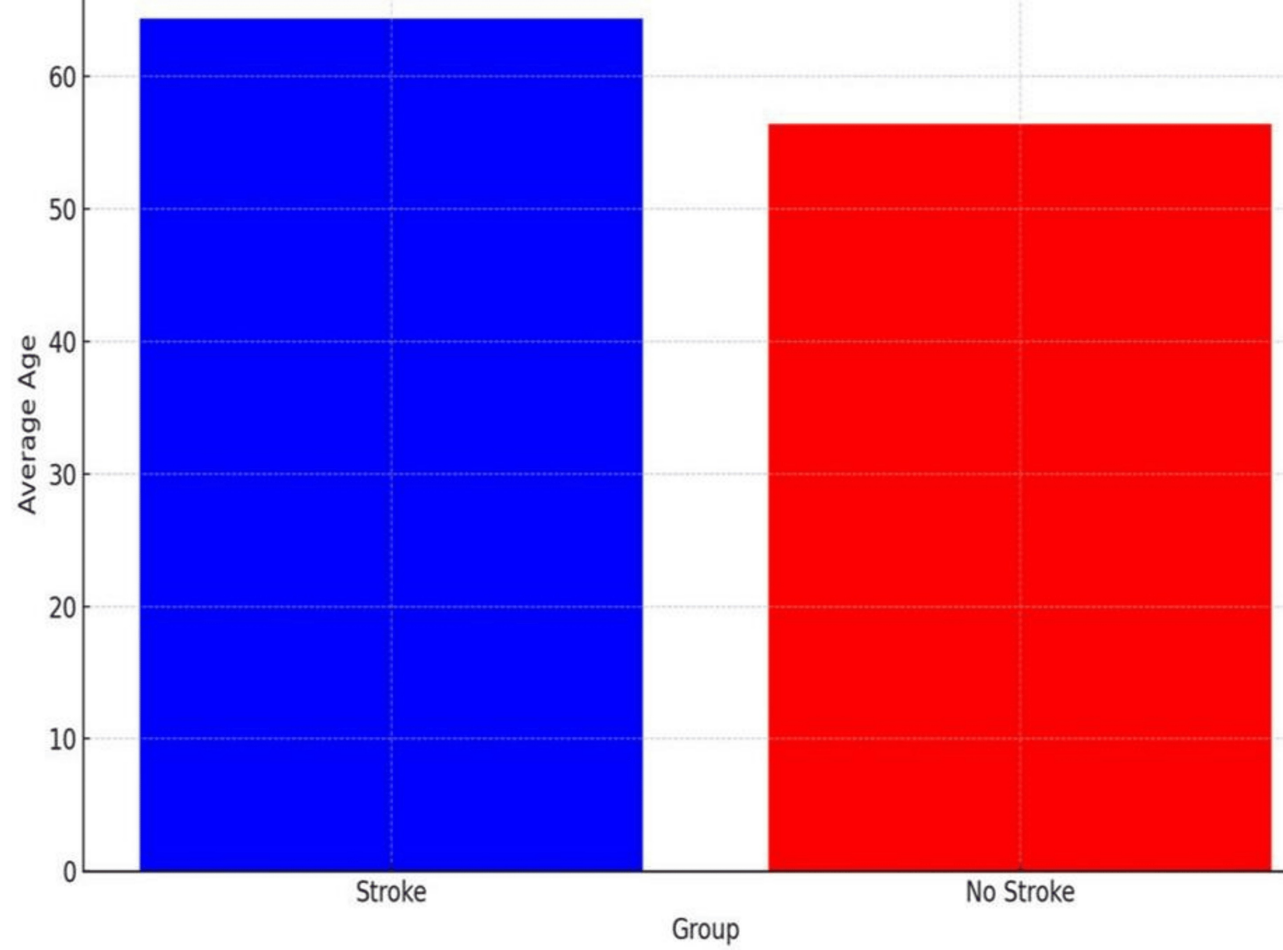
Mean age comparison between stroke and non-stroke patients

The bar chart reveals that patients with a history of stroke have an average age of 64.38 years, whereas patients without a history of stroke have an average age of 56.41 years (Figure 1).

**Figure 2:**
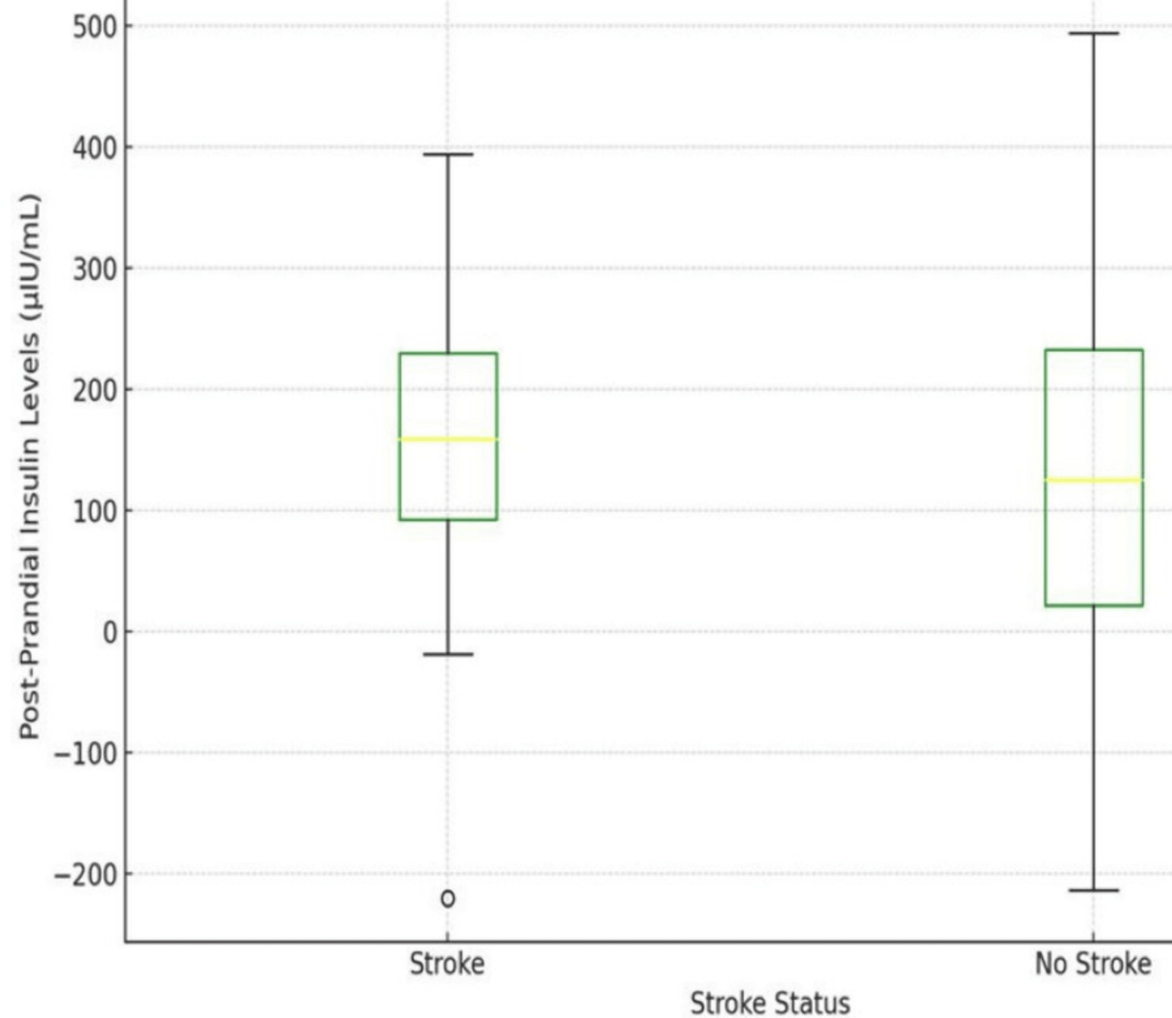
Comparison of postprandial insulin levels between stroke and non-stroke patients

The box plot represents the distribution of postprandial insulin levels in patients, comparing those with and without a history of stroke. The data indicates that the mean postprandial insulin level for stroke patients is 106.92 µIU/mL, while for patients without a stroke history, the mean is 128.2 µIU/mL. The chart highlights the disparity in post-prandial insulin levels among the two groups, suggesting a possible correlation between postprandial insulin levels and the incidence of stroke (Figure 2).

## Discussion

In our study, we performed a predictive analysis to investigate the impact of various factors on the occurrence of stroke. The variables most strongly related to stroke occurrence were age, fasting insulin, and postprandial insulin levels. An increase in fasting insulin levels was linked to a lower risk of stroke occurrence. On the other hand, increasing postprandial insulin levels and age were associated with an increased risk of stroke. Other factors such as FBS, PPBS, HbA1c, total cholesterol, TGL, HDL, LDL, VLDL, sex, and IHD had a relatively minor impact on the likelihood of participants experiencing a stroke. We found that the increase in the following factors may increase the rate of stroke occurrence: FBG, PPBS, HbA1c, HDL, LDL, and VLDL. However, interestingly, the increase in total cholesterol and TGL is associated with a minor decrease in the likelihood of stroke occurrence.

A previous meta-analysis by Xun et al. combined previous evidence to study the association between fasting insulin concentrations and the risk of hypertension, stroke, and IHD [14]. They reported that the higher the fasting insulin level, the higher the risk for hypertension and IHD [14]. However, they found that high fasting insulin levels have no role in increasing the likelihood of stroke, which is similar to our finding. This meta-analysis represents the most comprehensive synthesis of prospective cohort studies to date, examining the relationships between fasting insulin levels and the incidence of hypertension, stroke, and coronary heart disease (IHD).

Thacker et al. investigated the relation between insulin-resistant measures and ischemic stroke risk [15]. The study involved non-diabetic elderly individuals and followed them for 17 years for incident ischemic stroke. They found that the reduced Gutt insulin sensitivity index and increased level of two-hour glucose were linked to an increased risk of ischemic stroke. Surprisingly, elevated fasting insulin levels did not demonstrate a significant association with ischemic stroke.

The possible cause of the association between the Gutt index and two-hour glucose levels with stroke risk, while fasting insulin levels do not, may be attributed to the fact that peripheral insulin resistance might have a higher correlation with cardiovascular risk factors among older individuals. The postprandial measures of insulin resistance or glucose represent overall or peripheral insulin resistance because they give a combined picture of insulin response to oral glucose intake with uptake by skeletal muscle and fat tied to liver function. In contrast, fasting insulin levels primarily reflect hepatic insulin resistance, as they are influenced by liver activity. However, hepatic and peripheral insulin resistance tend to be interconnected, making it challenging to draw a clear distinction between them in epidemiological studies [17].

Another large study by Wieberdink et al. reported similar results to our study [18]. They explored the possibility that insulin resistance markers are associated with stroke risk in elderly people. The research involved 5,234 participants who were at least 55 years old without stroke and diabetes (1997-2001). Concentrations of fasting insulin and the homeostasis model assessment for insulin resistance were used as indicators of insulin resistance. To determine associations between insulin resistance markers and stroke risk, they performed Cox regression analysis while adjusting for age, sex, and potential confounders. During a median follow-up period of 8.6 years, there were 366 first strokes (comprised of cerebral infarctions, intracerebral hemorrhages, and unspecified stroke types). However, neither fasting insulin levels nor the homeostasis model assessment for insulin resistance showed significant associations with the risk of stroke or its subtypes in this non-diabetic elderly population.

On the other hand, previously published studies found a positive correlation between fasting measures of insulin resistance and the risk of ischemic stroke. The Atherosclerosis Risk in Communities (ARIC) Study, which involved participants without cardiovascular disease or diabetes at the baseline, found fasting insulin to be positively associated with ischemic stroke risk after adjusting for age, sex, race, and study site [19]. Similarly, these positive associations were also seen in smaller studies. This gap between our results and previous studies reporting the fasting insulin association with stroke risk could arise because people with a high fasting insulin level in previous studies could have had undetected diabetes [20,21].

Age is another significant risk factor for stroke. The risk for stroke doubles with each decade after the age of 50. The majority of strokes occur in individuals over the age of 65. The adult brain vasculature is highly complex, receives around 20% of the total cardiac output, and exchanges 20% of total blood glucose and oxygen. In the process of aging, there are structural and functional changes in both the micro- and macro-circulations in the brain. It can be assumed that age-related microcirculatory changes are caused by endothelial dysfunction and decreased brain autoregulation and neurovascular relations. Instead of endothelial dysfunction leading to neuroinflammation, impaired cerebral autoregulation causing microvascular injury, and impaired neurovascular coupling resulting in a decline in cortical function might all be strategies for future treatment. Aging (even in otherwise healthy individuals) often results in significant changes in the intracranial and extracranial cerebral arteries which can predict the future risk of stroke [22].

Russo et al. investigated the incidence of stroke in different age groups [23]. They analyzed the data from age groups over 80, between 80 to 84 years old, and over 85. The findings revealed a strikingly high incidence of strokes among the very old population. The estimated incidence rates were notably elevated, with individuals over 80 contributing almost a third of all strokes. Despite similar stroke rates across genders, those over 80 exhibited higher 30-day case fatality rates and dependency occurrences.

The limitations of our study may include residual confounding or bias, as systematic errors in measurement or unmeasured factors cannot be ruled out. Additionally, the small sample size cannot be excluded as a potential source of limitations. Hence, future research projects covering a larger sample size focusing on fasting insulin and its association with stroke risk should be implemented to address the scarcity of evidence available.

## Conclusions

In our study, we conducted a predictive analysis to explore factors influencing stroke occurrence. We found age, fasting insulin, and postprandial insulin to be the most influential variables. Higher fasting insulin levels were associated with a reduced risk of stroke, while increased postprandial insulin and age were linked to higher stroke risk. Other factors like blood glucose levels, cholesterol, and triglycerides had minor effects on stroke likelihood. Interestingly, higher total cholesterol and triglyceride levels were associated with a slight decrease in stroke occurrence. Further research with larger sample sizes is needed to validate these findings

## References

[1] Boehme AK, Esenwa C, Elkind MS (2017). Stroke risk factors, genetics, and prevention. Circ Res.

[2] Mozaffarian D, Benjamin EJ, Go AS (2016). Heart Disease and Stroke Statistics-2016 Update: a report from the American Heart Association. Circulation.

[3] Patne S, Chintale K (2016). Study of clinical profile of stroke patients in rural tertiary health care centre. Int J Adv Med.

[4] Ghotra SK, Johnson JA, Qiu W, Newton A, Rasmussen C, Yager JY (2015). Age at stroke onset influences the clinical outcome and health-related quality of life in pediatric ischemic stroke survivors. Dev Med Child Neurol.

[5] Kissela BM, Khoury JC, Alwell K (2012). Age at stroke: temporal trends in stroke incidence in a large, biracial population. Neurology.

[6] O’Donnell MJ, Chin SL, Rangarajan S (2016). Global and regional effects of potentially modifiable risk factors associated with acute stroke in 32 countries (INTERSTROKE): a case-control study. Lancet.

[7] Shehata GA, Abd-Elwahid L, Fathy M, Nasreldein A (2020). Prevalence of asymptomatic atherosclerosis of extracranial vessels among hypertensive patients in southern Egypt: an extracranial duplex study. Neurosciences (Riyadh).

[8] Kim YD, Cha MJ, Kim J (2011). Increases in cerebral atherosclerosis according to CHADS2 scores in patients with stroke with nonvalvular atrial fibrillation. Stroke.

[9] Lee EJ, Choi KH, Ryu JS (2011). Stroke risk after coronary artery bypass graft surgery and extent of cerebral artery atherosclerosis. J Am Coll Cardiol.

[10] Ali I, Abuissa M, Alawneh A (2019). The prevalence of dyslipidemia and hyperglycemia among stroke patients: preliminary findings. Stroke Res Treat.

[11] Murphy SJ, Werring DJ (2020). Stroke: causes and clinical features. Medicine (Abingdon).

[12] Cynthia A, Yogeesha KS, Arunachalam R (2014). Dyslipidemia in stroke. IOSR J Dent Med Sci.

[13] Palacio-Portilla EJ, Roquer J, Amaro S (2022). Dyslipidemias and stroke prevention: recommendations of the Study Group of Cerebrovascular Diseases of the Spanish Society of Neurology (Article in Spanish). Neurología.

[14] Xun P, Wu Y, He Q, He K (2013). Fasting insulin concentrations and incidence of hypertension, stroke, and coronary heart disease: a meta-analysis of prospective cohort studies. Am J Clin Nutr.

[15] Thacker EL, Psaty BM, McKnight B (2011). Fasting and post-glucose load measures of insulin resistance and risk of ischemic stroke in older adults. Stroke.

[16] Machin D, Campbell MJ, Tan SB, Tan SH (2009). Sample Size Tables for Clinical Studies.

[17] Matsuda M, DeFronzo RA (1999). Insulin sensitivity indices obtained from oral glucose tolerance testing: comparison with the euglycemic insulin clamp. Diabetes Care.

[18] Wieberdink RG, Koudstaal PJ, Hofman A, Witteman JC, Breteler MM, Ikram MA (2012). Insulin resistance and the risk of stroke and stroke subtypes in the nondiabetic elderly. Am J Epidemiol.

[19] Rasmussen-Torvik LJ, Yatsuya H, Selvin E, Alonso A, Folsom AR (2010). Demographic and cardiovascular risk factors modify association of fasting insulin with incident coronary heart disease and ischemic stroke (from the Atherosclerosis Risk In Communities Study). Am J Cardiol.

[20] Kernan WN, Inzucchi SE, Viscoli CM, Brass LM, Bravata DM, Horwitz RI (2002). Insulin resistance and risk for stroke. Neurology.

[21] Rundek T, Gardener H, Xu Q (2010). Insulin resistance and risk of ischemic stroke among nondiabetic individuals from the northern Manhattan study. Arch Neurol.

[22] Yousufuddin M, Young N (2019). Aging and ischemic stroke. Aging (Albany NY).

[23] Russo T, Felzani G, Marini C (2011). Stroke in the very old: a systematic review of studies on incidence, outcome, and resource use. J Aging Res.

